# The Rules of Being a Patient

**DOI:** 10.1007/s11606-025-09609-0

**Published:** 2025-05-20

**Authors:** Leah Ratner, Noor Elshaar

**Affiliations:** 1 Brigham and Women’s Hospital, Boston, MA USA; 2 Harvard Medical School, Boston, MA USA; 3 Patient partner, Boston, MA USA

Dear Noor,

Watching you stand on stage today—not untouched by suffering, but undefeated—I felt a mixture of pride, awe, and admiration. Your work spoke volumes, but more than that, it was witnessing your journey, knowing what it took to get here, that moved me most.

As you spoke about your cousins living with cystic fibrosis in Syria—walking the same path as you but denied basic resources—I was struck by the power of transforming pain into purpose.

I thought back to the last time we saw each other. It’s amazing how a day so routine for me—rounding on my patients—was a day that I know still haunts you. An unexpected transfer to the ICU. The moment we both knew what was next—a lung transplant.

I watched you then, the way I watched you now—with admiration for your poise, for your fierce ability to advocate, even in the face of uncertainty.

Lately, I’ve been struggling with my own health. The sicker I have been, the more I am paralyzed – afraid of speaking up and not being heard. The fear of advocating too much or not advocating enough. I aspire to be "the perfect patient"—but the unwritten manual of how to do that feels impossible.

Sincerely,

Leah

***Rule One:*** *Be polite, but not too polite. Ask for what you need, but not in a way that makes people uncomfortable. Show that you’re informed, but not in a way that threatens. Make sure they like you—because liked patients get better care.*

Dearest Leah,

Walking off that stage and seeing you push through the crowd, tears in your eyes, wrapping me in the warmest hug of the decade—I will cherish that moment forever. It was as if no time passed.

You are right. That day still haunts me. But talking about it with you makes it feel a little less heavy.

I, too, tried to be the “ideal” patient—never asking for too much, never showing too much emotion—because I worried that if I did, I wouldn’t be taken seriously.

Every breath I take since my transplant feels surreal. In honor of my donor, I’ve committed to giving back. In doing so, I’ve realized that we share something—a deep compassion for others. But compassion isn’t enough. It must be paired with accountability.

Breathe with me,

Noor

***Rule Two:*** *Do not let them see your fear. Fear makes you unreliable, emotional, difficult. Fear invites bias. The kind that makes people take a step back, question the validity of your pain. You have seen it happen, over and over again.*

Dear Leah,

Driving you to the airport that weekend wasn’t just a last-minute favor—it was a moment of healing. During those 28 minutes of soul-deep conversation, you asked me something that stopped me in my tracks: *"How do you not get paralyzed by fear in front of your doctors?"*

You told me about the times you advocated too hard and were labeled "difficult." The times you stayed silent—and were met with apathy, neglect, and medical error.

You wanted to know how I keep going. How I keep showing up, despite knowing that the way I present myself can determine the care I receive.

My answer was simple: I don’t. Some days, it feels impossible.

But what struck me most was realizing—even doctors have to “follow the rules” when they become patients. Even you have felt the consequences of apathy, bias, and neglect.

When I dropped you off, I wasn’t ready to say goodbye. I felt guilty that our shared medical trauma had forged friendship—but also profoundly grateful.

Breathe with me,

Noor

***Rule Three:*** *Stay on their good side. Be the kind of patient they enjoy taking care of. The one who makes them feel competent, kind, effective. A patient they will remember fondly—not as a problem to be solved.*

Dear Noor,

In medical school, we were taught that strict boundaries between doctors and patients were ethically necessary. But trusting each other enough to share our medical trauma—that taught me to question everything I had learned.

I didn’t know there were rules. Not until I felt that paralyzing fear—the fear that if I wasn’t perceived the right way, I wouldn’t get the care I needed.

We talk about the "hidden curriculum" in medical training—the unspoken biases that shape how we practice medicine. But I never realized the same is true for patients. There is an unspoken hierarchy of worth—an implicit expectation that some people deserve care more than others.

We are rewriting the rules. Medicine should be more than this.

Warmly,

Leah

***Rule Four:*** *Never make them feel like they made a mistake. Medicine is grey, and you know this better than most. You have been the doctor trying to fit a patient’s story into a framework. Discomfort is also where learning happens, and you are tired of holding the consequences of their blind spots alone.*

Dear Leah,

Today, I realized the rules don’t just exist between patients and doctors—they exist in the system itself.

Which brings me to–

***Rule Five:*** *Do not break the rules. Do not ask for too much. Do not inconvenience. Do not make them feel bad. Do not let things get worse. Do not let things go.*

But how do you follow all the rules at once?

You can’t.

And that is the problem.

We both know that medical bias—ableism, race, gender—dictates who gets care and who does not. We have both lived its consequences.

But what if we could change that?

What if we could take our experiences and use them to advocate for others?

What if our work became more than our stories—but a movement?

We have been patients, but we have also been on the other side.

And we know the rules.

But maybe, just maybe, it’s time to break them.

*Breathe with me,*

*Noor*

